# Supplementary material for: A Case of Persistent Diarrhea in a Man with the Molecular Detection of Various Campylobacter species and the First Isolation of candidatus Campylobacter infans
Source: Pathogens. 2020 Nov 30;9(12):1003. doi: 10.3390/pathogens9121003 (PMC7761484; doi:10.3390/pathogens9121003)
Supplement: Supplementary file 1 [file pathogens-09-01003-s001.pdf]

Table S1: Sequences of the *Campylobacter* species-specific qPCRs used in this report

Pathogen	Name	Label 5’	Sequence (5’ – 3’)	Quencher 3’	Reference
C. coli	Fccol1		TGACGGTAGAACTTTCAAATCC		[1], adapted
	Fccol2		ATGACTGTAGAACTTTCAAATCCTTA		
	Rccol		GCAAGTGCTTCACCTTCGATA		
	Pccol	6FAM	GAACAAATTCAAAAAACAGG	MGB-BHQ	
C. fetus	Fcfet2		TGAGGCTGTTACAAGCGAGTT		
	Rcfet2		CCACGATCAAACTGCATACCT		
	Pcfet2	VIC	TTCAGCAAATAGCGATAG	MGB-BHQ	
C. helveticus	Fchel		TGAAGCGATTGTTGATGAGC		[1], adapted
	Rchel		ACGCCATCTTTTCCAACTCTC		
	Pchel	NED	GTTGCGACAATTTCAGC	MGB-BHQ	
C. hyointestinalis	Fchyo		CTACGATCTCTGCAAATAGCGA		

	Rchyo1		ACCTTCAACAACAACAAGCTCA		
	Rchyo2		CCCTCAACAACAACAAGCTCA		
	Pchyo	6FAM	CTGAAGCTATGGAAAAAGTAG	MGB-BHQ	
C. jejuni	Fcjej		CTGGTGGTTTTGAAGCAAAGATT		[2]
	Rcjej		CAATACCAGTGTCTAAAGTGCGTTTAT		
	Pcjej	VIC	AATTCCAACATCGCTAATG	MGB-BHQ	
C. lari	Fclar		TTCTGCAAATTCAGATGAGAAAAT		
	Rclar1		CTCTATCAAATTGCATACCTTCAACTAC		
	Rclar2		CAAATTGCATGCCTTCAACTAC		
	Pclar	NED	AGTTGGAAAAGATGGTGTTAT	MGB-BHQ	
C. upsaliensis	Fcup		CGTTTTGGCACACGCTATTT		[1], adapted
	Rcup		CATCAACAATAGCCTCACAAGC		
	Pcup	NED	GCAGGAGCGAATCCTAT	MGB-BHQ	

Sequencing	C1112R	ACGTCGTCCACACCTTCCT	[3]

References:

[1] Chaban, B.; Musil, K.M.; Himsworth, C.G.; Hill, J.E. Development of cpn60-based real-time quantitative PCR assays for the detection of 14 Campylobacter species and application to screening of canine fecal samples. *Appl. Environ. Microbiol.* **2009***, 75*, 3055-3061, 10.1128/AEM.00101-09 [doi].

[2] de Boer, R.F.; Ott, A.; Guren, P.; van Zanten, E.; van Belkum, A.; Kooistra-Smid, A.M. Detection of Campylobacter species and Arcobacter butzleri in stool samples by use of real-time multiplex PCR. *J. Clin. Microbiol.* **2013***, 51*, 253-259, 10.1128/JCM.01716-12 [doi].

[3] Inglis, G.D.; Boras, V.F.; Houde, A. Enteric campylobacteria and RNA viruses associated with healthy and diarrheic humans in the Chinook health region of southwestern Alberta, Canada. *J. Clin. Microbiol.* **2011***, 49*, 209-219, 10.1128/JCM.01220-10 [doi].
